# *Reprimo* as a modulator of cell migration and invasion in the MDA-MB-231 breast cancer cell line

**DOI:** 10.1186/s40659-016-0066-7

**Published:** 2016-01-22

**Authors:** Kurt Buchegger, Carmen Ili, Ismael Riquelme, Pablo Letelier, Alejandro H. Corvalán, Priscilla Brebi, Tim Hui-Ming Huang, Juan Carlos Roa

**Affiliations:** Department of Pathology, Molecular Pathology Laboratory BIOREN-CEGIN, School of Medicine, Universidad de La Frontera, Temuco, Chile; School of Health Sciences, Universidad Católica de Temuco, Temuco, Chile; Centre for Translational Research in Oncology (CITO) and Department of Hematology and Oncology, Pontificia Universidad Catolica de Chile, Santiago, Chile; Department of Molecular Medicine/Institute of Biotechnology, University of Texas Health Science Center at San Antonio, STRF, Room 225, 7703 Floyd Curl Drive, San Antonio, TX 78229 USA; Department of Pathology, Advanced Center for Chronic Diseases (ACCDiS) (CITO), School of Medicine, Pontificia Universidad Católica de Chile, Santiago, Chile

**Keywords:** *Reprimo*, MDA-MB-231, Migration, Invasion

## Abstract

**Background:**

*Reprimo (RPRM)*, a highly glycosylated protein, is a new downstream effector of p53-induced cell cycle arrest at the G2/M checkpoint, and a putative tumor suppressor gene frequently silenced via methylation of its promoter region in several malignances. The aim of this study was to characterize the epigenetic inactivation and its biological function in BC cell lines.

**Methods:**

The correlation between *RPRM* methylation and loss of mRNA expression was assessed in six breast cancer cell lines by methylation specific PCR (MSP), 5′-Aza-2′-deoxycytidine treatment and RT-PCR assays. MDA-MB-231 cells were chosen to investigate the phenotypic effect of *RPRM* in cell proliferation, cell cycle, cell death, cell migration and invasion.

**Results:**

In the cancer methylome system (CMS) (web-based system for visualizing and analyzing genome-wide methylation data of human cancers), the CpG island region of *RPRM* (1.1 kb) was hypermethylated in breast cancer compared to normal breast tissue; more interesting still was that ERα(+) tumors showed higher methylation intensity than ERα(−). Downregulation of *RPRM* mRNA by methylation was confirmed in MDA-MB-231 and BT-20 cell lines. In addition, overexpression of *RPRM* in MDA-MB-231 cells resulted in decreased rates of cell migration, wound healing and invasion in vitro. However, *RPRM* overexpression did not alter cell viability, phosphatidylserine (PS) translocation or G2/M cell cycle transition.

**Conclusion:**

Taken together, these data suggest that *RPRM* is involved in decreased cell migration and invasion in vitro, acting as a potential tumor suppressor gene in the MDA-MB-231 cell line.

## Background

Breast cancer (BC) is the second most common cancer in the world and by far the most frequent cancer among women. Approximately, 1.67 million new cancer cases were diagnosed in 2012 (25 % of all cancers), being the second cause of cancer death in developed regions (198,000 deaths, 15.4 %) [1].

Loss of proliferation regulation and the activation of invasion and metastasis are considered hallmarks in many cancer types, including BC. These processes are tightly regulated within normal cells. A deregulation in proliferation may occur by several mechanisms, including direct expression of growth factor ligands and receptors by cancer cells or by induction of surrounding normal cells, which permits an uncontrolled proliferation of tumor cells. At this point, the role of tumor suppressor genes that limit growth and proliferation in cancer is important [2].

Cell migration plays a central role in a wide variety of biological phenomena in both normal physiology and pathophysiology. In a tumor setting, cell migration and invasion are the processes that allow malignant cells to change their position within tissue or between organs, penetrating tissue barriers such as the basement membrane, infiltrating the underlying interstitial tissue and spreading to the metastatic sites [3].

The associated cancer cells typically develop many alterations in morphology as well as in the cell–cell attachment and extracellular matrix to obtain the ability to dissociate intracellular adhesions and become motile. This process is usually driven by complex regulatory signaling cascades that temporarily and/or permanently alter the expression of several genes that reorganize the cytoskeletal network [2, 4, 5].

In 2000, Ohki et al. [6] identified a new gene called *Reprimo* (latin for repress), a potential new gene p53-dependent tumor suppressor by using differential display polymerase chain reaction (PCR) in x-ray-irradiated mouse embryonic fibroblasts.

*Reprimo* (*RPRM*) is located at 2q23 and encodes a highly glycosylated protein that shows four bands (16, 21, 23 and 40 kDa) found predominantly in the cytoplasm [6]. Nevertheless, in silico gene ontology analysis of its amino acid sequence show that RPRM is an integral component of cell membrane (URL: http://www.uniprot.org/uniprot/Q9NS64). Overexpression of *RPRM* by adenovirus transfection induces G2 arrest by inhibiting both Cdc2 activity and nuclear translocation of Cdc2-cyclin B1 complex in mouse embryonic fibroblast, acting as a suppressor of cell cycle progression. Cyclin B1, a key component in the control of cell cycle progression from G2 to M phase, has been implicated in tumorigenesis and the development of malignancy. Overexpression of cyclin B1 promotes cell invasive growth and extravasation through the capillary endothelium [7]. Therefore, *RPRM* acts as mediator of cell cycle transition, blocking nuclear transition of Cdc2-Cyclin B1 complex [6]. *RPRM* may repress cyclin B1-Cdc2 activity, promoting cell cycle arrest at the G2/M checkpoint and/or suppressing metastatic potential, exerting a tumor suppressive activity [7].

*RPRM* promoter methylation has been reported in several tumor cell lines and tumors including pancreas, head and neck, prostate, liver, gastric, renal and pituitary [8–16]. In gastric cancer, *RPRM* methylation has been detected frequently in plasma, promising to become a biomarker of the early stage of progression [13]. Furthermore, in esophageal cancer *RPRM* promoter methylation is significantly lower in chemoradiotherapy responders than in non-responders [17], and is predictive of a poor prognosis in pancreatic ductal carcinoma [10].

Nevertheless, in BC there have been no reports about whether *RPRM* mRNA levels are altered by promoter methylation and whether act as a repressive mechanism of mRNA expression, or about the functional significance of the ectopic expression of *RPRM* in the MDA-MB-231 cell line. Therefore, we decided characterize the epigenetic inactivation and its biological function of *RPRM* in BC cell lines.

## Results

### *Reprimo* is differentially methylated between BC and normal control sample tissues

**Fig. 1:**
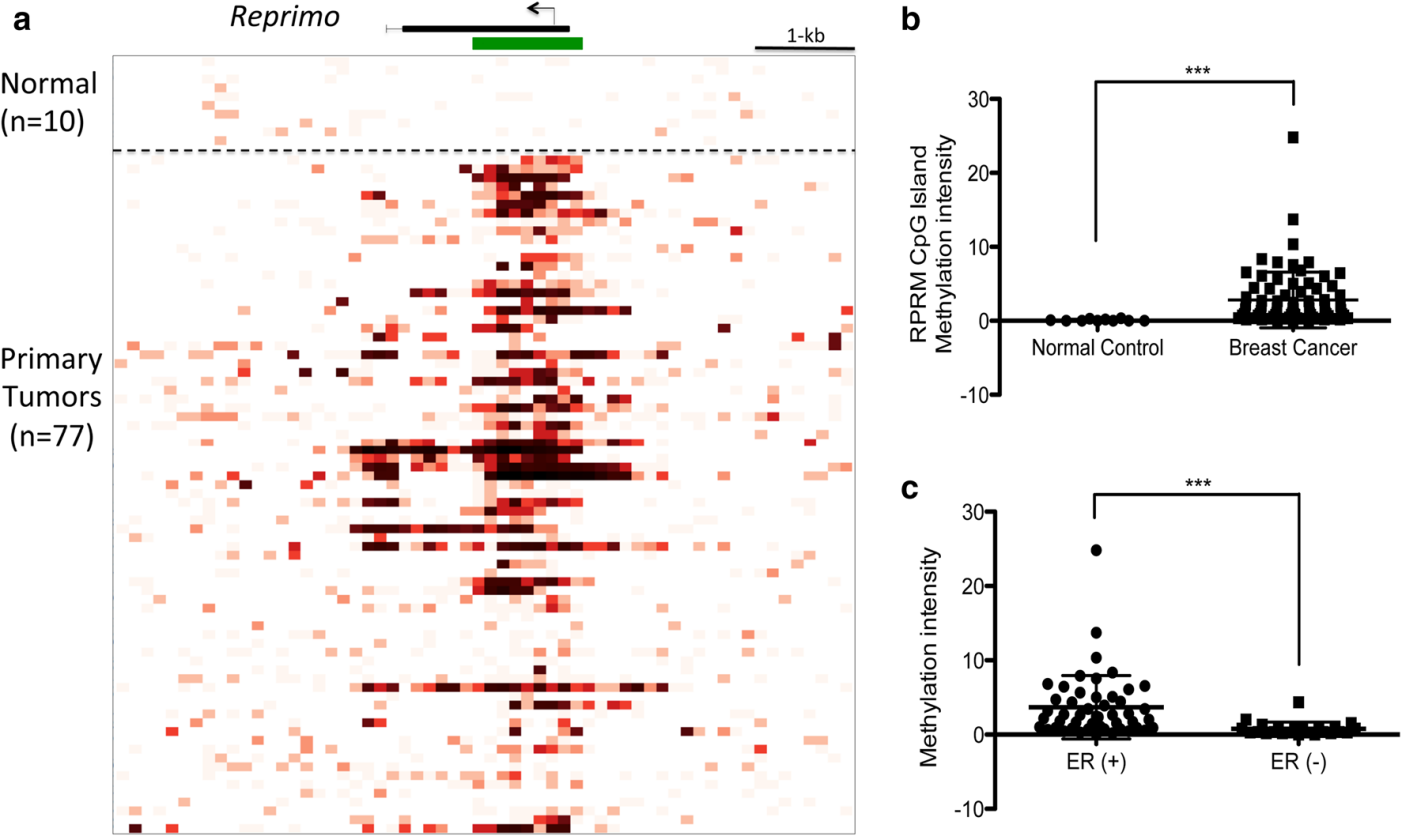
Differential methylation in *RPRM* CpG island between breast cancer and normal control samples. **a** Methylation intensity pre-calculated showing as a red gradient heatmap for *RPRM* CpG Island. At the *top* part of the figure; in *green*, shown CpG Island. In *black*, shown the gene body (from 153.992.097 to 154.093.568; Chromosome 2). The *black* arrow marks the start transcription site (TSS). The region analyzed corresponds to the CpG Island from 154.042.600 to 154.043.700 pb (length: 1.1 kb). The cases studied were ten normal breast (*top*) and 77 primary tumors (*below*). **b** Methylation intensity in BC was higher than normal control samples. **c**. Within BC group, ERα(+) group showed higher methylation intensity than ERα(−). The Mann–Whitney test was used for each analysis. The *error bars* indicate standard deviation. *P < 0.05, **P < 0.01, ***P < 0.001

The cancer methylome system (CMS) website uses a computational analysis to calculate the average intensity of *RPRM* CpG island methylation (Start–End: 154042600–154043700, length: 1.1 kb, Chromosome 2) between BC (77) and normal control samples (10) (Fig. 1a). The calculated methylation intensity was higher in the BC group than in the normal control group (Fig. 1b; P < 0.001). Moreover, we correlated the methylation data with clinic-pathological features, but there were no significant differences with any characteristic (data not shown). Nevertheless, when BC cases were classified into two groups: estrogen receptor positive [ERα(+)] and estrogen receptor negative [ERα(−)], we found higher methylation intensity in the ERα(+) group (Fig. 1c; P < 0.001). Unfortunately, we could not classify the BC tissues into other different molecular subtypes, such as Luminal A, Luminal B, Her2 and Basal-like, because data of the protein expression of Her2+ and Ki67 were incomplete. Based on these results, we decided to characterize the epigenetic inactivation and its biological function of *RPRM* in BC cell lines.

### *Reprimo* transcript is downregulated in BC cell lines by promoter methylation

To determine the methylation (M) or unmethylated (U) status of *RPRM* promoter, methylation specific polymerase chain reaction (MSP) technique was performed. The flanked region by primer in 5′-promoter region was described in previous reports [8, 18]. The primers were tested using 100 % methylated DNA and non-methylated DNA before to perform the experiment in cell lines (data not shown).

**Fig. 2:**
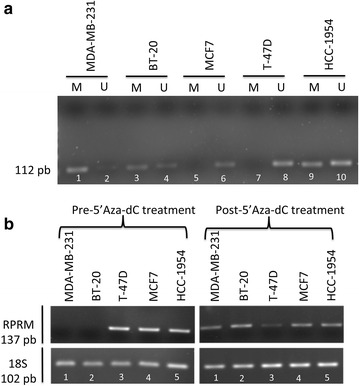
mRNA expression and promoter methylation of *RPRM* in breast cancer cell lines. **a** Aberrant methylation was found in 3/5 of breast cancer cell lines studied. *M* methylated status, *U* unmethylated status. PCR product = 112 pb. **b** Expression levels before and after treatment with 5′-Aza-dC, by RT-PCR. Cells were treated with 5′-Aza-dC (5 μM) for 5 days. In two (MDA-MB-231 and BT-20; *lane* 1 and 2, respectively) out of three BC cell lines the expression was decreased by methylation, and restored after 5′Aza-dC treatment. *RPRM* PCR product = 137; 18S PCR product = 102 pb

*RPRM* promoter was methylated in 3/5 (MDA-MB-231, BT-20 and HCC-1954) of the BC cell lines analyzed (Fig. 2a). Lost or repressed mRNA expression was found in 2/5 (MDA-MB-231 and BT-20) of BC cell lines (Fig. 2b). To validate MSP results, we performed a qMSP analysis, which revealed promoter methylation in MDA-MB-231 and BT-20, the same cell lines with a repressed *RPRM* expression (data not shown). After 5′Aza-dC treatment, *RPRM* mRNA expression in MDA-MB-231 and BT-20 was restored (Fig. 2b). Therefore, we concluded that the transcriptional silencing of *RPRM* due to its promoter methylation in BC.

### *Reprimo* overexpression suppresses cell migration and invasiveness in MDA-MB-231

**Fig. 3:**
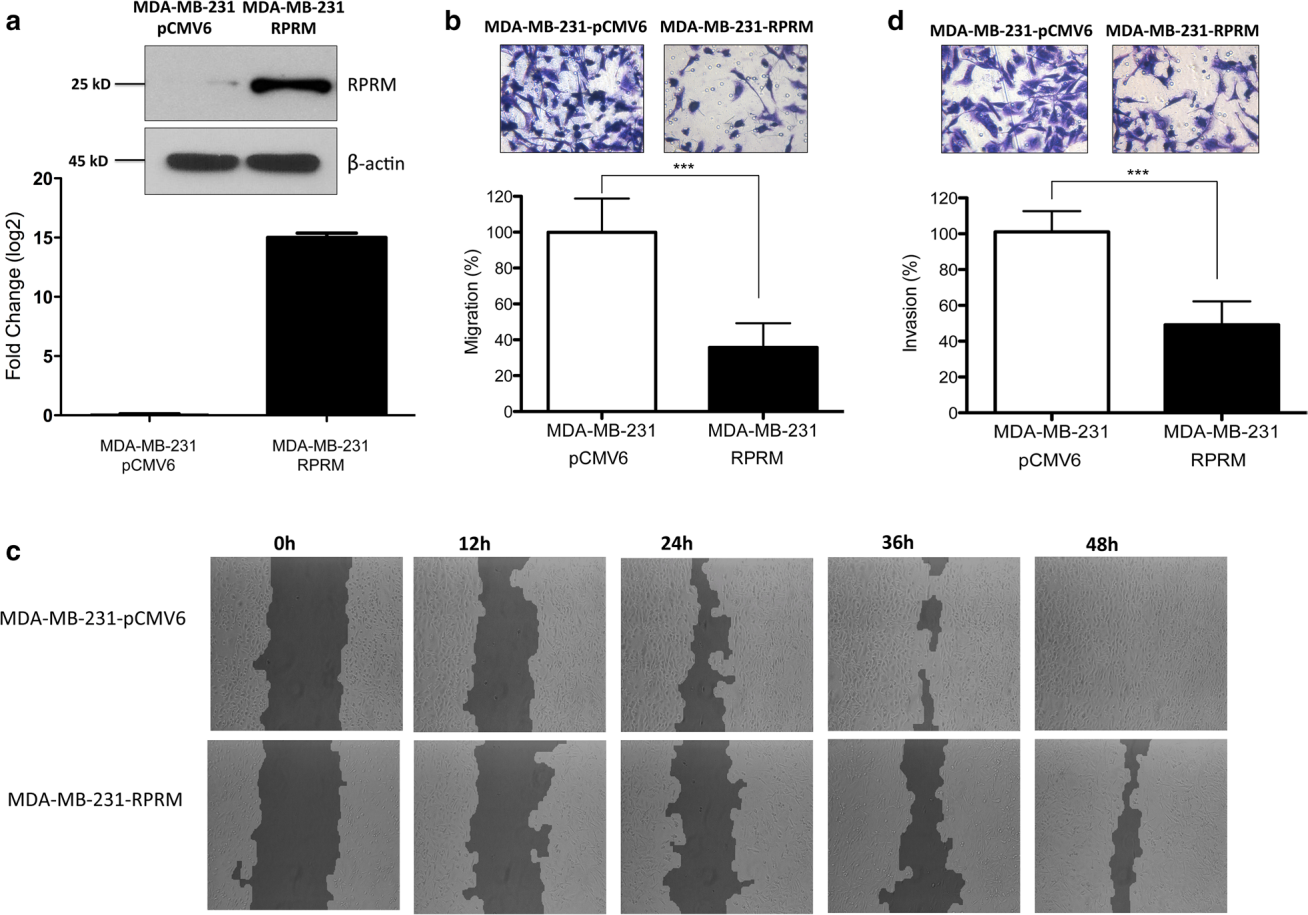
Effect of *RPRM* overexpression on migration and invasion properties in MDA-MB-231 cells. **a** qPCR and Western Blot of MDA-MB-231 cells with overexpression of RPRM (MDA-MB-231-RPRM) and empty control (MDA-MB-231-pCMV6). The antibody used was a monoclonal antibody anti-DDK. Reprimo protein (12kD) is detected as 23–25 kD approximately due to glycosylation. **b** Overexpression of *RPRM* in MDA-MB-231 cells negatively regulates the migration ability. Representative micrographs of transwell migration assay were selected to show each condition (40× magnification). Quantification of cell migration expressed as the percentage of control. **c** Overexpression of *RPRM* in MDA-MB-231 reduced wound healing ability. Representative micrographs were taken at 0, 12, 24, 36 and 48 h (10× magnification). **d** Overexpression of *RPRM* in MDA-MB-231 cells significantly reduced the invasion at 24 h using a matrigel-coated insert. Representative images of invaded cells were selected to show each condition (40× magnification). Quantification of cell invasion expressed as the percentage of control. For each assay values were compared to control (MDA-MB-231-pCMV6) using a Mann–Whitney test. The *error bars* indicate standard deviation. *P < 0.05, **P < 0.01, ***P < 0.001

MDA-MB-231 cells were transiently transfected either with pCMV6-empty or with pCMV containing the full-length cDNA of *RPRM* gene (pCMV-RPRM). MDA-MB-231 cells transfected with pCMV6-RPRM (MDA-MB-231-RPRM cells) showed an increase in mRNA and protein levels of RPRM, compared to pCMV6-empty (MDA-MB-231-pCMV6 cells) (Fig. 3a). We tested whether *RPRM* could alter cell migration and invasion by wound healing assay and assays based in boyden chamber with or without matrigel matrix. Results in transwell migration assays showed a decrease of MDA-MB-231-RPRM cells migration in about 64.3 % (35.7 % of migrated cells) compared to MDA-MB-231-pCMV6 (Fig. 3b; P< 0.0001). To verify these results, we performed a wound-healing assay. On this regard, we found that 48 h post-scratch, the wound was completely closed in MDA-MB-231-pCMV6 cells, while in MDA-MB-231-RPRM cells the wound remained open (Fig. 3c). To study the invasiveness potential of these treated cells, we perform an invasion assay using a Boyden chamber coated with matrigel. We found that effectively MDA-MB-231-RPRM cells migrated 51 % less than MDA-MB-231-pCMV6 cells (Fig. 3d; P < 0.0001). In summary, transfected cells with RPRM-containing vector (MDA-MB-231-RPRM) exhibited significantly lower cell migration and invasion rate than those transfected with empty vector (MDA-MB-231-pCMV6), confirming for first time that RPRM regulates cell migration and invasion in cancer cells, particularly in BC cells.

### *Reprimo* overexpression failed to induce cell death and cell cycle arrest in MDA-MB-231 BC cells

**Fig. 4:**
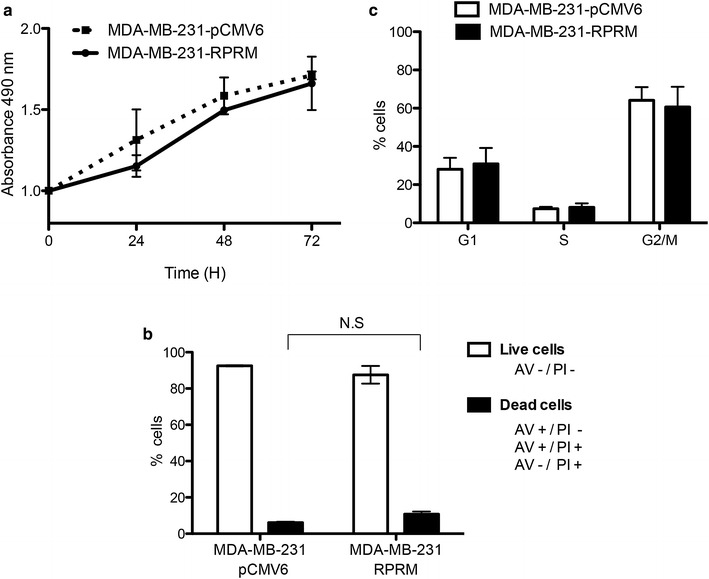
Role of *RPRM* in cell death and cell cycle arrest in MDA-MB-231 cells. **a** Overexpression of *RPRM* in MDA-MB-231 cells did not decrease cell viability. **b** Ectopic expression of *RPRM* did not significantly increase cell death in MDA-MB-231 cells. **c** Modulating *RPRM* levels had no effect on progression of cell cycle phases. MDA-MB-231-pCMV6 was used as a control. For each experiment, RPRM mRNA and protein levels were confirmed by qPCR and Western blot with an anti-flag antibody, respectively

There were no statistical differences in cell viability between cells expressing either a control vector (MDA-MB-231-pCMV6) or *RPRM* (MDA-MB-231-*RPRM*) (Fig. 4a). To determine the functional consequences of *RPRM* overexpression in regard to programmed cell death, an Annexin V (AV)/propidium iodide (PI) staining assay was performed in MDA-MB-231 cells at 48 h post-transfection. Overexpression of *RPRM* slightly increased apoptosis and cell death, but these differences were not significant in compared with control-transfected cells (Fig. 4b). Flow cytometry analyses were grouped into three types of cell stages according to the Nomenclature Committee on Cell Death (2012): live cells (AV−/PI−), apoptotic cells (AV+/PI−) and death cells (AV+/PI+, and AV−/PI+). The average percentage for apoptotic cells was 4.0 % for *RPRM* and 2.5 % for control cells. The average percentage for death cells was 6.8 % for *RPRM* and 3.7 % for control cells. In summary, in terms of apoptotic cells and death cells, we confirmed that cell viability was not significantly decreased in MDA-MB-231-*RPRM* compared with MDA-MB-231-pCMV6.

Cells were synchronized by serum deprivation for cell cycle profiles; however, there were no changes in the percentages of cells in the G2/M phase between MDA-MB-231-pCMV6 and MDA-MB-231-*RPRM* cells (Fig. 4c). Therefore, RPRM does not have a significant effect to alter G2/M in MDA-MB-231 cells.

## Discussion

*Reprimo* is a potential p53-dependent tumor suppressor, located at 2q23 and encodes a highly glycosylated protein, located predominantly in the cytoplasm. It is believed to regulate the activity of the Cdc2-cyclin B1 complex by interfering with an as yet unknown checkpoint G2/M mechanism operating in the cytoplasm [6]. Several studies have shown that tumor suppressor genes that participate in cell cycle arrest are frequently inactivated through aberrant methylation in human malignances [19]. In this regard, *RPRM* promoter methylation has been found in several human cancers, including pancreas [8, 10, 20], head and neck [9], esophagus [17, 21], prostate [11, 22], lung [23], kidney [16], pituitary [14] and gastric [13, 15, 24]. There have been no reports regarding methylation status or functional studies in BC. Using the CMS platform [25], we analyzed the methylation status of the *RPRM* CpG island region (1.1 kb) in BC tissues, finding hypermethylation in cancer tissue but not in normal control samples. Moreover, methylation intensity was higher in ERα(+) than ERα(−) in the BC group, suggesting that *RPRM* methylation is linked to ER status. Similarly, Malik et al. proposed a repression of *RPRM* gene transcription mediated by a tripartite interaction between ERα, histone deacetylase 7 (HDAC7), and FoxA1 [26]. We decided characterize the epigenetic inactivation and its biological function of *RPRM* in BC cell lines.

MSP was performed on five BC cell lines, which revealed methylation of the promoter region of *RPRM* in 3/5 (MDA-MB-231, BT-20 and HCC-1954) lines analyzed. However, qMSP analysis showed promoter methylation in 2/5 lines (MDA-MB-231 and BT-20; data not shown). These two lines exhibited loss or repression of *RPRM* mRNA expression. When we treated these cell lines with 5′Aza-dC, mRNA expression was restored, indicating that methylation is the main mechanism of gene silencing of *RPRM* in the MDA-MB-231 and BT-20 cell lines. This phenomenon was also observed in esophageal adenocarcinoma and squamous carcinoma cell line OE33 and KYSE 110 [21], respectively, and in three renal cell carcinoma cell lines (KTCL26, SKRC18 and SKRC39) [16]. Interestingly, *RPRM* is located at chromosome 2q23.3, a locus that often has allelic imbalance in BC [27]; nevertheless, we observed that loss or repression of mRNA expression was restored by 5′Aza-dC treatment, concluding that there is probably no allelic imbalance of 2q23 involved in loss of *RPRM* mRNA expression in these two cell lines.

Interestingly, only two cells lines (BT-20 and MDA-MB-231) showed *RPRM* promoter methylation with mRNA expression loss, both of them ERα(−) cells. Surprisingly, in ERα(+) cells MCF7 and T47D the *RPRM* promoter was hypomethylated and presented mRNA expression in contrast to ERα(+) BC tissues results, likely because cells culture are not being exposed to estrogen as BC tissues.

In functional studies, we tested whether *RPRM* could be less viable and increase PS translocation in MDA-MB-231 cells, as previously reported in gonadotrope and GH pituitary cells [14]. However, there were no differences between cells with *RPRM* and the empty control. Also, we enquired as to whether *RPRM* could act as a G2/M phase cell cycle brake in MDA-MB-231 BC cells as previously reported in mouse embryonic fibroblast [6]. Unfortunately, *RPRM* cannot significantly alter G2/M in MDA-MB-231 cell line. Our results of cell cycle arrest are consistent with those in pituitary [14] and gastric tumors [15], suggesting that *RPRM* may perform an alternative cell function in non-fibroblast derived cells, or even depending on tumor type.

In addition, we examined whether *RPRM* has a role in cell migration and invasion. In an immunohistochemistry study involving S100A2 and RPRM, Luo et al. found that loss of RPRM protein expression was significantly correlated with the depth of tumor invasion, lymphatic vessel invasion, and lymph node metastasis, suggesting tumor suppressor activity of RPRM in human clinical gastric tumor tissue [28]. In our study, we expressed *RPRM* ectopically in MDA-MB-231 cells, finding a significant decrease in cell migration by transwell inserts and wound healing as well as a significant decrease in cell invasion by inserts with matrigel, supporting an important role in cell mobility and invasion in MDA-MB-231 cells.

These results are not consistent with previous results about the biology of *RPRM*. However, a previous study by Song et al. into cyclin B1 (a protein target of *RPRM*) demonstrated that, in addition to its known role in arresting the G2/M cell cycle phase, overexpression of cyclin B1 in human esophageal squamous cell carcinoma (ESCC) promotes cell invasive growth and extravasation through the capillary endothelium. Furthermore, in xenograft mice, overexpression of cyclin B1 is able to enhance lung metastasis in ESCC. Likewise, suppression of endogenous cyclin B1 inhibits the metastatic potential of ESCC to the lung. In addition, cyclin B1-induced ESCC metastasis appears to be linked to alteration of epithelial mesenchymal transition (EMT) molecules [7]. Given that *RPRM* regulates the activity of the Cdc2-cyclin B1 complex, it is likely that the antagonist effect in cell migration and invasion is through cyclin B1 inactivation.

## Conclusion

Our study was the first to report on the possible role of *RPRM* in the modulation of cell migration and invasion process in vitro in BC. Further investigation into the *RPRM*-regulated molecules is required, to clarify the mechanisms by which *RPRM* could exert tumor suppressive activity in migration and invasion capability.

## Methods

### Cancer methylome system analysis

The CMS is a web-based database application designed for the visualization, comparison and statistical analysis of human cancer-specific DNA methylation. The BC database was constructed with 77 breast tumors, 10 normal breast samples (mammary reduction) and 41 BC cell lines. The datasets were obtained with the MBDCap-seq protocol, a technique used to capture methylated DNA by using a methyl-CpG binding domain (MBD) protein column followed by next-generation sequencing [25, 29]. We performed comparative analyses of DNA methylation profiles between normal controls and BC samples, which were downloaded from the CMS. Then, by computational analysis the average intensity correlation of methylation of *RPRM* CpG Island (1.1 kb length) was calculated in these two groups. BC tissue samples were fresh frozen tissue with >70 % tumor cellularity.

### Cell lines and transfections

BC cell lines MDA-MB-231, BT-20, T-47D, MCF7 and HCC1954 were generously provided by Dr. Tim Hui-Ming Huang (University of Texas Health Science Center at San Antonio, TX). MDA-MB-231 and BT-20 were cultured in Dulbecco’s Modified Eagle’s medium (high glucose), T-47D and HCC1954 in Roswell Park Memorial Institute (RPMI) 1640 medium (Thermo Scientific HyClone, Logan, UT, USA) and MCF7 in Advanced Modified Eagle’s medium (Invitrogen, Life Technologies corporation, Grand Island, NY, USA) supplemented with 10 % fetal bovine serum, 10 units/mL of penicillin and 10 mg/mL streptomycin (1 % penicillin/streptomycin, Thermo Scientific Hyclone). All five cell lines were incubated at 37 °C in a humidified atmosphere containing 5 % CO_2_ and subculture during the logarithmic phase.

To generate MDA-MB-231 transient-transfections, cells were transfected with pCMV6-*RPRM*-Myc-DDK-tagged or pCMV6 (empty vector control) (OriGene Technologies, Inc), using Lipofectamine 2000 (Invitrogen) according to the manufacturer’s instructions. All experiments were carried out in technical and biological triplicate. The success of transfection was confirmed by quantitative real-time PCR and western blot.

### 5′-Aza-2′-deoxycytidine treatment

Breast cancer cell lines were incubated in culture medium with the demethylating agent 5-Aza-2′-deoxycytidine at a concentration of 5 μM for 5 days. Fresh media were added at 24 and 72 h. Cells were harvested and RNA was extracted at day 5 [16].

### Real time PCR

Total cell RNA was isolated from cells using the TRIzol reagent (Life Technologies) according to the manufacturer’s instructions. First-strand cDNA was prepared from 1 μg of cell RNA in a total reaction volume of 20 μL using M-MLV reverse transcriptase 200 U/μl (Promega Corp., Madison, WI) at 42 °C for 60 min. The resulting cDNA was subsequently amplified by PCR using the Brilliant II Ultra-Fast SYBR^®^ Green qPCR Master Mix according to the manufacturer’s protocol on the Stratagene Mx-3000p real-time PCR system (Agilent Technologies Inc., Santa Clara, CA). Relative fold levels were determined using the 2^−ΔΔCT^ method, with 18S used as housekeeping control. The primer pairs used are detailed in Table 1.

**Table 1:** Primer and probe sequences used in this study

ID	Sequences (5′–>3′)	PCR product (pb)	References
*RPRM* (forward)	CTGCGAATTTGAACGGGGTGAGT	137	–
*RPRM* (reverse)	GCGTAAACCGTGCAGTCACGA	137	–
*RPRM*-M (forward)	GCGAGTGAGCGTTTAGTTC	120	Sato et al. [8]
*RPRM*-M (reverse)	TACCTAAAACCGAATTCATCG	120	Sato et al. [8]
*RPRM*-U (forward)	TTGTGAGTGAGTGTTTAGTTTG	113	Sato et al. [8]
*RPRM*-U (reverse)	TAATTACCTAAAACCAAATTCATC	113	Sato et al. [8]
B-actin-M (forward)	TGGTGATGGAGGAGGTTTAGTAAGT	133	Moon et al. [30]
B-actin-M (reverse)	AACCAATAAAACCTACTCCTCCCTTAA	133	Moon et al. [30]
*RPRM* (probe qMSP)	/56-FAM/TT CGC GTC G/ZEN/T TCG CGG CGT TCG TT/3IABkFQ/	120	–
B-actin (probe qMSP)	/56-FAM/AC CAC CAC C/ZEN/C AAC ACA CAA TAA CAA ACA CA/3IABkFQ/	133	Moon et al. [30]

*M* methylated, *U* unmethylated

### DNA extraction

Genomic DNA from cells was extracted using phenol/chloroform and absolute ethanol DNA precipitation. The quantity and quality of extracted DNA were assessed by measuring the samples in a NanoDrop 1000 spectrophotometer (Thermo Fisher Scientific Inc., Waltham, MA).

### Sodium bisulfite modification

A total of 1 μg of genomic DNA was modified with sodium bisulfite using the EZ DNA Methylation™ Bisulfite Kit (Zymo Research, Irvine, CA) following the manufacturer’s protocol. Bisulfite-converted DNA was stored at −80 °C until use.

### Methylation-specific PCR (MSP)

*RPRM* promoter was amplified from bisulfite-converted DNA using specific primers for methylated and unmethylated DNA, obtained from a previous report (Sato N et al. [8]). Primer sequences and annealing temperature are provided in Table 1. Bisulfite-modified genomic DNA was amplified by PCR, the cycle of which was: (a) 95 °C for 5 min; (b) 35 cycles: 95 °C 30 s, 59 °C 30 s, and 72 °C 30 s; (c) 72 °C 5 min for a final extension. As a positive control 100 % methylated DNA was used (Zymo Research, Irvine, CA). PCR mix without DNA was used as a blank. PCR products were analyzed by electrophoresis in 1.5 % agarose gel and visualized by gel red staining (Biotium, Hayward, CA) under UV light.

### Quantitative methylation-specific PCR (qMSP)

To quantify the methylation level of *Reprimo*, quantitative methylation-specific PCR (qMSP) was performed. Amplification reactions were carried out in triplicate in a volume of 20 μL that contained 1 μL of bisulfite-modified DNA; 300 nM of each primer; 50 nM probe (Table 1); 0.375 U platinum *Taq* polymerase (Invitrogen); 100 μM of dNTPs; 100 nM ROX dye reference (Invitrogen); 8.3 mM ammonium sulfate; 33.5 mM Trizma (Sigma, St. Louis, MO); 3.35 mM magnesium chloride; 5 mM mercaptoethanol; and 0.05 % DMSO [30]. Amplifications were carried out using the following profile: 95 °C for 10 min followed by 40 cycles at 95 °C for 30 s, 59 °C for 30 s and 72 °C for 30 s. Amplification reactions were carried out in 96-well plates in the Mx3000P QPCR System (Stratagene). Each plate included DNA samples, positive control (100 % methylated DNA, Zymo Research) and water blanks. The relative level of methylated DNA for *RPRM* was determined as a ratio of methylation-specific PCR-amplified gene to β-actin (reference gene) and then multiplied by 1000 for easier tabulation (average value of triplicates of the study gene divided by the average value of triplicates of β-actin × 1000) [30, 31].

### Western blot

2 × 10^5^ MDA-MB-231 cells were transiently transfected with pCMV6-*RPRM*-Myc-DDK-tagged or pCMV6 (empty vector control) (OriGene Technologies, Inc), using Lipofectamine 2000 (Invitrogen) according to the manufacturer’s instructions. Cells were harvested and whole-cell lysates were extracted using RIPA buffer (50 mM Tris, pH 7.2; 150 mM NaCl; 1 % Triton X-100; and 0.1 % SDS).

With Protease and Phosphatase Inhibitor Cocktail Kit (Sigma Aldrich). Protein concentrations were determined using Pierce BCA protein assay kit (Pierce, Thermo Fisher Scientific Inc, Rockford, IL, USA) according to the manufacturer’s instructions. Equal amounts of total cellular protein (40 μg) were separated by sodium dodecyl sulfate–polyacrylamide gel electrophoresis in 4–12 % NuPAGE^®^ Bis–Tris Precast Gels (Novex, Life Technologies Corporation) and electrotransferred to polyvinylidene difluoride membranes (Immobilon^®^-P membrane, Millipore, Bedford, MA, USA). The membranes were blocked with 1 × Tris-buffered saline containing 0.05 % Tween (TBST) and 5 % fat-free milk for 1 h at room temperature. Primary antibodies anti-DDK (Origene technologies; cat. Nº TA50011) and anti β-actin (Cell Signalling; cat.Nº 13E5) were diluted 1:2,000 and 1:5,000, respectively, in TBST/3 % BSA, the membranes were incubated in primary antibodies at 4 °C overnight. The Membranes were washed three times in TBST for 10 min. Both anti-mouse and anti-rabbit peroxidase-conjugated secondary antibody (Santa Cruz) were diluted in 1:20,000 in TBST 1X and incubates the membrane for 1 h at room temperature. The membranes were washed as above and visualized using SuperSignal West Pico (Pierce) according to the manufacturer’s protocol.

### Viability assays

Cell viability in transfected MDA-MB-231 cells was determined using the CellTiter 96 AQueous one solution (Promega). Five thousand MDA-MB-231cells were seeded in a 96-well plate (four replicates for each condition) and allowed to attach overnight. Transfection was performed 24 h later, using Lipofectamine 2000 (Invitrogen) according to the manufacturer’s instructions. Cells were incubated for 24, 48 and 72 h after transfection. Control wells contained only cell-free medium. Twenty microliters of CellTiter MTS solution were added to each well, and plates were incubated for 1 h at 37 °C. Absorbance was measured at 490 nm.

### Apoptosis analysis

To study cell death from within transfected MDA-MB-231 cells to the outer leaflet of the plasma membrane, we used the Alexa Fluor 488 Annexin V/Dead Cell Apoptosis Kit (Life Technologies), staining according to the manufacturer’s instructions, and analyzed it by flow cytometer (FACs Canto II, Becton–Dickinson). A total of 2.5 × 10^5^ MDA-MB-231 cells were plated in a 6-multiwell plate (two replicates of each condition) and allowed to attach overnight. Transfection was performed 24 h later, using Lipofectamine^®^ 2000 (Invitrogen) following the manufacturer’s protocol. Cells were incubated for 48 h post-transfection. Positive control cells were treated with 5 % DMSO for 24 h. Wild-type cells were used as a negative control.

### Cell cycle analysis

MDA-MB-231 (2.5 × 10^5^ cells per well) cells were plated in a 6-multiwell plate (two replicates of each condition) and allowed to attach overnight. Cells were synchronized by starvation in serum-free media for 24 h. Immediately, transient transfection was performed using Lipofectamine^®^ 2000 (Invitrogen). Cells were incubated for 48 h post-transfection in complete media. After incubation period, the cells were trypsinized, re-suspended, fixed (ethanol 70 % for 2 h at 4 °C) and finally stained with PI solution (0.1 % (v/v) Triton X-100, 10 μg/mL PI, and 100 ug/mL DNase-free RNase A in PBS). Cell cycle profiles were gathered using a Flow cytometer (FACs Canto II, Becton–Dickinson) at the Universidad de La Frontera Scientific and Technological Bioresource Nucleus (BIOREN).

### Cell migration

A total of 5 × 10^4^ transient MDA-MB-231 transfected cells were suspended in 300 μl of serum-free DMEM medium and seeded into the upper chamber of each insert (24-well insert; pore size, 8 μm; BD Biosciences). Then, 500 μL of DMEM containing 10 % FBS were added to a 24-well plate. After incubation at 37 °C for 12 h, migrated cells were washed (twice with DPBS), fixed (Methanol 100 %) and stained for 15 min in crystal violet solution (0.5 % crystal violet in 25 % methanol/DPBS). Cells that did not migrate to the lower compartment were removed with a cotton swab. Each insert was photographed in five random fields at a magnification of 40×. Quantification is expressed as the percentage of area covered with migrated cells by using ImageJ software (Wayne Rasband, National Institute of Health, USA).

### Wound healing

A total of 1 × 10^5^ MDA-MB-231 cells were plated in a 24 multi-well plate. Transient transfection was performed after 24 h. Cells were allowed to form a confluent monolayer in a 24-well plate before wounding. A sterilized pipette tip was used to generate wounding across the cell monolayer, and the debris was washed with PBS. Cells migrating into the wounded area were visualized and photographed under an inverted microscope at varying intervals. A total of six areas were selected randomly in each condition and photographed at a magnification of 10×.

### Cell invasion

MDA-MB-231 cells were transfected with pCMV6-*RPRM* or pCMV6 vector using Lipofectamine 2000 (Invitrogen), following the manufacturer’s protocol. Serum-induced cell invasion were performed at 37 °C for 24 h using a 24-well transwell insert (24-well insert; pore size, 8 μm; BD Biosciences) coated with 30 μg of matrigel (BD Biosciences). 5 × 10^4^ cells suspended in 200 μl serum-free medium were seeded into the upper chamber and 600 μl complete medium into the lower chamber. After 24 h, the upper surface of the insert was wiped gently with a cotton swab to remove non-migrating cells. Cells that migrated and invaded through the membrane were stained with crystal violet solution (0.5 % crystal violet in 25 % methanol/DPBS), and photographed by a microscope with a camera in five random fields at a magnification of 40×. Quantification is expressed as the percentage of area covered with migrated cells by using ImageJ software (Wayne Rasband, National Institute of Health, USA).

### Statistical analysis

Data were analyzed by a Mann–Whitney test, Kruskal–Wallis test and two-way ANOVA with Bonferroni’s multiple comparisons test using GraphPad Prism software (San Diego, CA). Values were expressed as mean ± SD. Values of P < 0.05 were considered statistically significant.

### Additional data

The data supporting the result about *RPRM* methylation in BC tissues and cell lines were obtained from CMS website available in http://cbbiweb.uthscsa.edu/KMethylomes/.

## Abbreviations

*RPRM*: *Reprimo*
CMS: cancer methylome system
PCR: polymerase chain reaction
RT-PCR: retrotranscriptase polymerase chain reaction
qPCR: quantitative polymerase chain reaction
BC: breast cancer
ER: estrogen receptor
PR: progesterone receptor
Her2: human epidermal receptor 2
MSP: methylation specific polymerase chain reaction
qMSP: quantitative methylation specific polymerase chain reaction
MTS: 3-(4,5-dimethylthiazol-2-yl)-5-(3-carboxymethoxyphenyl)-2-(4-sulfophenyl)-2H-(tetrazolium) inner salt
PS: phosphatidilserine
